# Artificial Feeding Systems for Vector-Borne Disease Studies

**DOI:** 10.3390/biology13030188

**Published:** 2024-03-15

**Authors:** Olayinka M. Olajiga, Samuel B. Jameson, Brendan H. Carter, Dawn M. Wesson, Dana Mitzel, Berlin Londono-Renteria

**Affiliations:** 1 Department of Tropical Medicine and Infectious Disease, Tulane University, New Orleans, LA 70112, USA; sbishop@tulane.edu (S.B.J.); bcarter5@tulane.edu (B.H.C.); wesson@tulane.edu (D.M.W.); 2 Animal Diseases Research Unit, National Bio- and Agro-Defense Facility, United States Department of Agriculture, Agricultural Research Service, Manhattan, KS 66506, USA; dana.mitzel@usda.gov

**Keywords:** artificial feeding system, vector-borne diseases, VBDs, vector biology

## Abstract

**Simple Summary:**

Artificial feeding systems have emerged as a vital tool in research on arthropods like mosquitoes, ticks, blackflies, sandflies, tsetse flies, fleas, and triatomine bugs, aiding in the understanding of pathogen transmission. This review explores various artificial feeding systems used to study human–vector relationships and pathogen transmission, detailing their roles in insect-related research. We discuss the advantages and disadvantages of these systems, their practical applications, and speculate on future directions in vector-borne disease research. Recognizing the strengths and weaknesses of different artificial feeding systems will help researchers to choose the right tools for developing effective pathogen transmission and disease control strategies.

**Abstract:**

This review examines the advancements and methodologies of artificial feeding systems for the study of vector-borne diseases, offering a critical assessment of their development, advantages, and limitations relative to traditional live host models. It underscores the ethical considerations and practical benefits of such systems, including minimizing the use of live animals and enhancing experimental consistency. Various artificial feeding techniques are detailed, including membrane feeding, capillary feeding, and the utilization of engineered biocompatible materials, with their respective applications, efficacy, and the challenges encountered with their use also being outlined. This review also forecasts the integration of cutting-edge technologies like biomimicry, microfluidics, nanotechnology, and artificial intelligence to refine and expand the capabilities of artificial feeding systems. These innovations aim to more accurately simulate natural feeding conditions, thereby improving the reliability of studies on the transmission dynamics of vector-borne diseases. This comprehensive review serves as a foundational reference for researchers in the field, proposing a forward-looking perspective on the potential of artificial feeding systems to revolutionize vector-borne disease research.

## 1. Introduction

Vector-borne diseases (VBDs) are caused by pathogens that are transmitted to humans through the bites of infected arthropods like mosquitoes, ticks, and sandflies. These diseases continue to be a major global health concern, affecting millions annually and placing a substantial burden on public health [1,2,3,4]. Among them, malaria and dengue alone contribute to approximately 400,000 and 40,000 annual deaths, respectively [4]. Other VBDs, such as chikungunya fever, Zika fever, yellow fever, West Nile fever, and several neglected diseases also impact millions worldwide [1,4].

The transmission of VBDs relies on the interplay between vectors, pathogens, and hosts. It begins with an infected vector feeding on a susceptible host and depositing the pathogen into the host’s skin [5]. Once inside the host, the pathogen may replicate and disseminate through the host’s tissues. This process is influenced by factors like the host’s immune response and pathogen virulence [6,7]. The transmission cycle may then be sustained by the infected host serving as a source of infection for the vector. This highlights the complex interactions among vectors, pathogens, and hosts, emphasizing the multifaceted nature of vector-borne disease dynamics.

The study of these complex VBDs systems has relied heavily on live animal or human feedings [8,9,10,11,12]. While vertebrate animals offers several advantages, such as providing a natural physiological environment for the arthropods and offering insights into host–pathogen interactions [13,14], ethical and practical concerns exist [8,14,15,16,17]. Alternative methods including artificial feeders have emerged to address some of these limitations and provide a more controlled approach [18,19,20,21,22]. Artificial feeding systems incorporate advanced tailored membranes, capillary feeding mechanisms, and engineered biocompatible constructs to enhance experimental accuracy and support arthropod colonies in laboratory settings [23].

Artificial feeding aligns with the three Rs: the principles of Replacement, Reduction, and Refinement. The first refers to the replacement of animal use, the second refers to reduction by minimizing the number of animal use, and the third encompasses the refinement realized by enhancing experimental procedures to minimize any potential pain or distress experienced by the animals [24]. These systems emulate natural feeding conditions, allowing for precise observations and analyses of vector behaviors during blood feeding [25,26,27]. Artificial systems not only reduce inherent variability in live animal experiments but also offer enhanced insights into vector behaviors, transmission dynamics, and intervention strategies [28].

The controlled environment provided by artificial feeding systems allows for the exploration of a wider range of experimental conditions. While these systems attempt to mimic natural conditions, they also have inherent limitations. Therefore, the aim of this review is to explore and compare various artificial feeding systems and their advantages and limitations.

## 2. Use of Vertebrate Hosts to Study Arthropod Feeding and Pathogen Transmission

The study of VBDs often involves the use of vertebrate animals such as mice, rats, non-human primates (NHPs), and birds. Vertebrate animals facilitate investigations into various aspects of pathogen transmission, including feeding mechanisms in immune and non-immune hosts, and the potential for pathogen transmission based on the timing of feeding [29,30,31].

Early explorations in the research of VBDs were focused on understanding the transmission cycles and infection mechanisms of pathogens [32,33]. These foundational investigations have shaped subsequent research endeavors, emphasizing the role of animal models in deepening understanding and informing intervention strategies. However, studies have observed considerable variability in vector feeding patterns across different animal hosts [34,35,36,37,38,39,40,41,42,43,44,45,46]. This variability can be influenced by the specific characteristics of the vector species involved, including their feeding preferences, number of vectors per animal, life cycle, and physiology.

**Animal models**: Mouse models have been extensively used to explore various facets of VBDs, including the timing and quality of blood meals transmitted by *Anopheles stephensi* mosquitoes in malaria transmission [47]. Similarly, mouse models have contributed insights into the transmission dynamics of Zika virus and Mayaro virus via *Aedes aegypti* mosquitoes [36,48], *Borrelia burgdorferi* transmission by *Ixodes* spp. [49], and the acquisition dynamics of Tick-Borne Langat Virus in *Ixodes scapularis* [50]. Non-human primates (NHPs), particularly rhesus macaques, exhibit genetic and immunological similarities to humans, making them valuable models for studying immune responses, disease progression, and therapeutic evaluations [51,52,53]. Studies on Chagas disease and Yellow fever virus showcase the relevance of NHPs in closely replicating human disease progression [53,54], despite challenges in handling and monitoring, the need for specialized facilities, ethical considerations, and cost implications [55]. A number of birds, such as the Eurasian blackbird, house sparrow, American robin, and domestic chicken, have provided information about the interactions between viruses like St. Louis encephalitis virus and West Nile virus and their reservoir hosts [39,56,57,58,59].

The use of animal models in scientific studies offers several valuable advantages [Table 1], such as enabling the study of physiological processes and disease mechanisms [14], providing widely available and standardized animal models that offer a consistent platform for research, and granting flexibility in genetic manipulations to meet specific study requirements [13]. Despite these advantages, animal models present some limitations and ethical considerations. These models may not fully replicate human physiology and can create challenges in translating findings to clinical applications [60]. Ethical considerations regarding animal welfare and the use of anesthesia in vector feeding experiments have prompted discussions, calling for the continual reassessment and potential refinement of research methodologies [61,62,63]. Also, maintaining animal colonies and conducting experiments can be resource-intensive in terms of both time and cost [64].

**Human Challenge**: Human challenge studies, also known as controlled human infection models (CHIMs), represent a unique approach to studying VBDs. Unlike scenarios where human volunteers primarily aid in sustaining vector colonies, CHIMs involve deliberately exposing volunteers to arthropods infected with attenuated pathogens [65,66]. The historical backdrop of VBDs such as malaria and yellow fever traces back to the 19th and early 20th centuries, when patterns of disease transmission and associated symptoms from vector bites were meticulously documented through CHIMs [67]. Over time, the understanding of these diseases progressed, culminating in more formalized research methodologies in the mid-20th century.

Ethical considerations have played a significant role in the evolution of human challenge studies. In the latter half of the 20th century, the establishment of ethical guidelines and regulatory frameworks, including documents like the Nuremberg Code and the Declaration of Helsinki, highlighted the importance of informed consent and the protection of research participants [68]. In modern times, human challenge studies have evolved to incorporate sophisticated monitoring systems, genetic analysis, and modeling approaches. These advancements have allowed for deeper insights into disease dynamics and more effective evaluations of interventions.

This intentional exposure induces mild disease, allowing for a structured investigation into the feeding behaviors and transmission dynamics of various VBDs [69,70]. CHIMs play a pivotal role in advancing the comprehension of vector–host interactions, thereby facilitating the development of robust disease control measures [71]. CHIMs also provide insights into the transmission dynamics of diseases like dengue and malaria [72,73]. Notably, CHIM studies have significantly contributed to examining clinical inflammation in malaria-naïve volunteers and devising effective malaria control strategies [74,75]. CHIMs have been instrumental in unraveling the transmission dynamics of the Leishmania parasite [76,77,78]. Given the inherent risks associated with these infections, stringent safety protocols are rigorously enforced to safeguard the well-being of participants. 

The advantages of using CHIMs in research on VBDs include their ability to facilitate highly controlled studies, promoting detailed investigations into disease mechanisms and immune responses [79]. CHIMs provide realistic outcomes and highly relevant data by accurately reflecting the complexities of human physiology, immune responses, and disease interactions [80]. CHIMs help maintain temperature homeostasis, crucial for optimal vector feeding and pathogen development, and preserve natural odor cues that guide vector behavior during feeding. CHIMs have emerged as indispensable tools for evaluating vaccine candidates against a spectrum of diseases, including malaria, dengue fever, Leishmania, and Chagas disease [76,77,78,81,82,83,84,85,86,87]. However, CHIMs present ethical considerations, including potential risks to participants and the need for stringent safety protocols [88]. Variability in disease severity among individuals participating in CHIMs can affect the generalizability of findings to broader populations [89] [Table 1]. Despite these challenges, CHIMs stand as a valuable tool in advancing our understanding of VBDs.

## 3. Artificial Feeding Systems for Vectors

Progress in vector-borne disease research is enhanced by advancements in feeding methods by mimicking natural feeding platforms to provide insights into feeding behaviors and the competence of vectors as disease carriers [12,23]. These systems vary in complexity, from basic setups using synthetic and animal hide membranes to advanced constructions utilizing individual capillary feeding or biocompatible constructs.

**Artificial Membrane Feeding:** The least complex artificial feeding systems utilize membranes that emulate natural feeding surfaces [23]. While artificial membranes offer insights into vector-borne diseases, their universal applicability can be limited, as some vectors do not interact with them. Careful membrane selection has made artificial membrane feeding successful with a wide range of species. Semi-artificial feeding systems employing animal hides and intestines have gained attention for simulating natural feeding environments, especially for specific vectors like ticks and sandflies [22,90,91,92]. Parafilm M and silicon are two widely synthetic biocompatible materials used for artificial feeding membranes, closely mirroring natural host tissues [22,23,93,94,95]. These materials ensure that membranes do not elicit harmful immune responses, making them effective tools for vector-borne disease studies. Collagen membranes and polytetrafluoroethylene (PTFE) membranes are also used in constructing artificial feeding membranes for vectors [96,97,98]. Membrane feeding has proven valuable across various vector species, including mosquitoes, ticks, and tsetse flies [99,100,101,102].

A specialized in vitro artificial membrane system is the Hemotek feeding system, allowing for precise controlled blood feeding of hematophagous insects in the laboratory [21,97]. Equipped with electronic temperature controls and a blood reservoir, this system regulates the feeding environment to mimic natural conditions [21]. The Hemotek feeding system is relevant for the standardization of the infectious agent dose, vector competence, and pathogen transmission studies [103,104,105]. It has been instrumental in various pathogen transmission studies, such as studies on malaria transmission [106], evaluating the effectiveness of different blood sources for rearing *Ae. aegypti* [107], *Leishmania* spp. infections in sandflies [108,109,110], xenomonitoring in tsetse flies [111,112], investigating *Trypanosoma cruzi* in triatomine bugs [113,114,115], and examining *Yersinia pestis* in fleas [116]. Hemotek feeders have the advantage of temperature control, standardization, and commercial availability [106]. However, Hemotek feeders present drawbacks, such as their high cost and the need for regular maintenance [117].

Another in vitro artificial feeding system is the glass feeding device, commonly known as the Rutledge-style feeder [118]. Constructed with glass chambers, these devices allow arthropods to feed on a membrane or artificial surface [26,119]. Similar to the Hemotek feeder, odors or chemical cues derived from hosts can be introduced into the feeders to further enrich the feeding process and heighten attraction [120]. Glass feeders find extensive applications in studying mosquito feeding behavior, host preferences, tick feeding patterns, sandfly establishment and maintenance, virus transmission, and various infections in sandflies and fleas [121,122,123,124,125,126,127,128,129,130,131,132,133,134,135]. 

Like the Hemotek feeding system, glass feeding devices maintain controlled environmental conditions closely resembling natural settings. The transparent glass construction facilitates the observation of the feeding process, allowing for the collection of valuable data on vector behavior. These devices offer the advantage of enabling repeated feeding attempts, enhancing experimental flexibility and robustness. Despite these advantages, the use of glass can complicate biosafety protocols, limiting their suitability for certain studies. Their widespread use in research introduces a distinct challenge associated with Rutledge-style assays that utilize animal pelts as membranes for certain vectors. These pelts serve as a natural and biologically relevant substrate for tick and flea studies but may introduce challenges in maintaining consistency, ensuring reproducibility, and exercising experimental control [121,136,137]. The utility of glass feeders is further constrained due to the inherent fragility of glass material [106]. 

The use of artificial membrane feeding systems in vector-borne disease studies offers distinct advantages over animal or human feeding. They minimize reliance on live animal hosts, addressing ethical concerns and reducing overall animal use in research [138,139]. Artificial feeding membranes provide a controlled and standardized feeding environment, improving the accuracy and reliability of experimental results. They enable the precise observation and analysis of vector behaviors during feeding, contributing to a deeper understanding of vector biology, pathogen transmission dynamics, and potential intervention strategies. The benefits and ethical considerations associated with artificial feeding membranes highlight their significance as valuable tools in vector research. Artificial feeding membranes facilitate oral infection studies, offering flexibility in blood sources (cow, avian, or human blood, etc.) and enabling the collection of vector saliva for further analysis [140]. They are cost-effective, easy to use, and versatile [141]. 

However, one of the primary concerns with artificial feeding membranes is the potential for altered vector behavior due to variations in vector feeding conditions. Some studies have indicated that feeding behaviors and infection rates might differ between vectors feeding on artificial membranes compared to natural hosts [116,142,143,144], which could influence the reliability of experimental outcomes [Table 1]. The primary challenge in employing artificial membranes for tick research is mimicking the complex feeding environment encountered by ticks during host attachment, as they secrete a combination of saliva and cement-like substances to facilitate attachment and blood feeding, involving intricate biochemical interactions and structural adaptations [145]. Sandflies, with their very short mouthparts, may face difficulties in feeding on artificial devices. Therefore, it is crucial to choose suitable membranes such as those derived from animal skin or intestine to accurately replicate the unique feeding mechanisms and environmental conditions relevant to specific vectors.

**Capillary Feeding**: More specialized than membrane feeding devices are capillary feeding systems, offering an alternative artificial feeding approach specifically designed for certain hematophagous insects [146]. In this system, vectors like ticks feed through a capillary tube filled with a blood substitute or animal/human blood. Capillary feeding has been applied in various tick species to explore aspects of tick biology and pathogen transmission, such as the relationship between the rickettsia pathogen *Anaplasma marginale* and the tick species *Dermacentor variabilis* [19]; the infection of nymphal *Ixodes scapularis* ticks with *Borrelia burgdorferi,* the causative agent of Lyme disease [147]; establishing a laboratory colony of *Rhipicephalus (Boophilus) microplus* (formerly *Boophilus microplus*) ticks [148]; and evaluating the potential of selected tick proteins as antigens for reducing cattle tick infestations and infections with *Anaplasma marginale* and *Babesia bigemina* pathogens [149]. This has also been employed to assess the transmission of *Leishmania* spp. in sandflies [150] and investigate the vector competence of sandflies [146].

Capillary feeding offers finer control over individual vectors, thereby standardizing the feeding process [150]. But the limitation of capillary feeding in mimicking natural feeding behaviors may restrict its broader applicability in certain experimental contexts, such as studies involving tick cement. Capillary feeding might not be suitable, as ticks typically initiate feeding by partially engorging on hosts to create cement cones [145,151]. This critical step cannot be adequately replicated using capillary tubes. The suitability of capillary feeding can vary based on the specific vector and species involved.

The primary application of capillary feeding in mosquitoes involves the controlled collection of insect saliva for vector-borne pathogen studies [152,153]. This method is considered invasive for mosquitoes, often involving immobilization by removing their legs and wings to facilitate saliva collection into capillary tubes [152]. The fine and controlled nature of capillary tubes for collecting minute quantities of saliva is essential for downstream analyses requiring sensitivity, such as pathogen detection or the identification of bioactive compounds [152]. The temporal control offered by this method allows for saliva collection at specific time points [154], providing insights into dynamic changes in saliva composition over the course of feeding and shedding light on how vector-borne pathogens are transmitted to hosts.

**Engineered Biocompatible Constructs:** These are artificial materials carefully designed to replicate the structure and properties of the mammalian skin [18,155,156]. The constructs consist of crosslinked polymer networks with the ability to absorb and retain substantial amounts of water, imparting a soft, flexible, and gel-like structure that is similar to host skin [155,157,158,159,160,161]. These characteristics render them a viable alternative for delivering blood meals in vector-borne pathogen studies, closely mirroring the natural feeding conditions experienced by vectors in the wild [156]. The integration of engineered human tissue into these biocompatible constructs has facilitated investigations into the mechanical characteristics of the skin and the activities of vectors, notably mosquitoes, during feeding processes [18,155].

Recent advancements in mosquito feeding behavior studies have seen the emergence of two innovative systems utilizing engineered biocompatible constructs. One system employs bio-printed vascularized skin mimics designed to replicate natural feeding conditions, establishing a controlled environment for in-depth investigations into mosquito feeding behavior [18]. This platform demonstrated its capabilities through the successful evaluation of repellent effects, positioning it as a promising tool for future repellent screening assays. In parallel, another approach focused on the intricacies of arthropod bite-site biology. This study utilized tissue engineering techniques to create a Biologic Interfacial Tissue-Engineered System (BITES) designed to mimic the human dermal microvascular bed. These engineered constructs, cellularized with specialized cell types, showcased the potential for detailed analyses of vector–host–pathogen interactions [155]. Both systems represent significant contributions, expanding the capabilities of mosquito research. While the bio-printed vascularized skin mimics platform provides a controlled setting to explore mosquito feeding behavior and repellent efficacy, the BITES platform presents a novel approach to investigate complex interactions at arthropod bite sites. Together, these innovations highlight the transformative potential of engineered systems in advancing the understanding of vector biology and disease transmission dynamics.

Similar to other artificial feeding systems, the use of engineered biocompatible constructs provides an ethical alternative to methods relying on live animals or human volunteers [Table 1]. The benefits include the capacity to emulate the native feeding environment of vectors within an adjustable framework [18]. In contrast to other artificial feeding systems, engineered biocompatible constructs replicate the mechanical and physical attributes of human skin, offering a more precise simulation of the innate feeding mechanism [162]. This precision is crucial in studying pathogens transmitted by vectors, as understanding the natural feeding process is essential in determining the conditions most favorable for pathogen transmission, providing an accurate and realistic environment for the study of transmission, infection, and pathogenesis. The strength of bioengineered biocompatible constructs lies in their ability to function as versatile cell culture platforms, seamlessly integrating with living cells and tissues [155]. This inherent biocompatibility creates an optimal environment for the examination of cellular interactions. Engineered biocompatible constructs can also be tailored to mimic different skin types and conditions [163].

By providing a controlled and reproducible environment, this technique facilitates the study of host immune responses to vector feeding [164,165]. The utilization of engineered biocompatible constructs also enables investigations into pathogen entry mechanisms [166,167], providing greater control over the feeding process. This control extends to simulating different skin types and regulating blood flow rates within the gel [18], enhancing precision in the study of disease transmission dynamics. Despite their potential advantages, engineered biocompatible constructs come with notable limitations. This technique, while coming the closest to accurately simulating the host’s disease manifestation, may still fall short of capturing all the intricacies and complexities seen in natural systems. Biocompatible constructs often have a finite lifespan [168], potentially compromising the longevity of experimental results. Another significant drawback is the substantial cost associated with designing, producing, and maintaining such advanced systems [169]. The successful implementation and interpretation of experiments using these constructs necessitate a high level of technical expertise, further adding to the complexity and potential challenges in their use. 

**Table 1:** Overview of the advantages and limitations of various vector feeding systems.

Feeding Systems	Advantages	Limitations	References
Animal models	Practicality of use Physiological and genetic similarity to humans Availability Flexibility to genetic manipulations	Biological variations between animals and humans Ethical considerations Resource- and cost-intensive Variability in vector feeding patterns	[8,9,10,14,32,33,34,35,36,37,38,39,40,41,42,43,44,45,46,55,60,61,62,64,163,164,165]
Human challenge studies	Realistic outcomes Maintenance of temperature homeostasis and cues Controlled environment	Ethical considerations Variability in disease severity among individuals Potential risk to participants	[65,66,71,76,79,80,85,86,87,88,89]
Artificial membrane feeding	Controlled feeding Facilitate oral studies and the collection of saliva from vectors Eliminates ethical concerns Flexibility in blood sources Easy observation of vector feeding Easy to use, readily available, convenient, and cost-effective Reproducibility	Limited realism compared to natural hosts Variability in vector feeding behaviors Challenges in mimicking specific feeding mechanisms and conditions Animal pelt membrane limitations Biosafety protocols for glass feeders High cost Need for technical expertise	[23,107,117,118,141,142,143,144]
Capillary feeding	Precise control and standardization Easy collection of vector saliva Easy observation of vector feeding Eliminates ethical concerns	Limited to certain vectors Blood volume available is relatively small compared to a live host. Technical complexities Invasive for mosquitoes	[146,149,150,151,152,153,154,170,171]
Engineered biocompatible constructs	Close simulation of natural conditions Eliminates ethical concerns Biocompatibility with living cells and tissues Controlled and reproducible environment Flexibility to mimic different skin types Easy collection of vector salivary components	Limited realism Limited lifespan due to degradation High cost Need for technical expertise	[18,155,156,162,163,164,165,166,167,168,169]

## 4. Application of Artificial Feeding Systems 

**Vector competence studies:** The evaluation of a vector’s ability to competently transmit a pathogen to a host is a multifaceted process influenced by various factors, including the vector’s physiological responses to the ingested pathogen [172,173]. Artificial feeding systems play a crucial role in this evaluation, allowing for assessing infection rates, replication kinetics, and dissemination patterns within vectors by introducing specific pathogen quantities into artificial blood meals [174,175]. The adaptable nature of artificial feeding setups facilitates the examination of factors influencing vector competence, such as temperature and nutritional influences [176,177,178]. These systems serve as a valuable platform for assessing the efficacy of novel control strategies, including vector control measures and vaccinations [179]. Continuously evolving and improving, these methods significantly contribute to the understanding of vector competence intricacies, informing strategies for combating VBDs.

In studies focusing on pathogen acquisition, artificial feeding systems have been instrumental in investigating the complex mechanisms of vector–pathogen interactions. They provide a means to explore vector susceptibility factors, including immunological responses, environmental influences, and vector genetics, all of which impact the vector’s ability to acquire and transmit pathogens [180,181]. Artificial feeding systems also enable the precise control of vector infection status, facilitating the evaluation of various factors’ effects on transmission rates [182]. Such studies are pivotal for understanding the factors driving VBD transmission, including the effects of pathogen load and the duration of the infectious period [23]. 

**Drug and Vaccine Development:** Artificial feeding methods have emerged as a promising frontier in the development of drugs and vaccines against VBDs. This innovative approach provides a controlled platform for evaluating the efficacy of pharmaceutical compounds and vaccine candidates within a vector–host–pathogen context [183,184,185]. Live attenuated vaccine candidates such as DDVax are administered to mosquito vectors using artificial feeding systems, mimicking their natural feeding behaviors [185]. The setup facilitates investigations into the vaccines’ effects on pathogen transmission and vector competence. 

Artificial feeding presents significant opportunities in evaluating novel drug candidates targeting VBDs. Vectors can be exposed to controlled doses of antiparasitic or antiviral compounds via artificial blood meals, enabling the assessment of drug efficacy in reducing pathogen load within vectors [186]. By identifying compounds that impede pathogen development within vectors, these methods hold the potential to disrupt the transmission cycle of VBDs. As artificial feeding techniques continue to advance, they have the capacity to expedite the discovery of novel interventions against VBDs, bolstering global efforts to combat these formidable health challenges.

## 5. Future Directions for Artificial Feeding Systems

**Advancements in Biomimicry:** Recent advancements in biomimicry herald a new era of precision and relevance in vector-borne disease studies, offering highly efficient artificial feeding systems that closely emulate natural feeding mechanisms. Innovative biomimetic surfaces have been developed, closely mimicking the complex topography and physiological cues of natural host tissues [18,155]. By replicating the texture, chemical composition, and even temperature of host skin, these surfaces create a more realistic feeding experience for vectors [156]. This approach is hypothesized to induce immunological responses in both vectors and biomimetic artificial feeding systems closely resembling those occurring in natural feeding environments. Advancements in biomimicry extend beyond surface properties, potentially incorporating factors like pH gradients, mechanical resistance, and localized temperature variations to create feeding environments more similar to natural conditions. Such biomimetic approaches hold the potential to unravel unique aspects of vector biology and pathogen transmission that have proven elusive using other methods.

**Microfluidics and Nanotechnology:** The integration of microfluidics and nanotechnology marks a transformative paradigm shift in artificial feeding systems for vector-borne disease studies. These cutting-edge technologies provide the ability to engineer microenvironments mirroring host conditions with unprecedented precision. Microfluidic devices enable the controlled delivery of feeding solutions [184], facilitating investigations into vector–pathogen interactions at a cellular and molecular scale [187]. By reproducing dynamic aspects of blood feeding, such as shear forces and blood pressure, these devices offer a more accurate representation of natural feeding conditions. Nanotechnology further enhances these systems, enabling the design of surfaces with tailored properties [188] that influence vector behaviors and physiological responses. Nanoscale textures, chemical gradients, and controlled release mechanisms can be harnessed to manipulate vector interactions with pathogens and feeding solutions. This combination of microfluidics and nanotechnology holds the potential to dissect the minute mechanisms governing pathogen acquisition and transmission, thereby opening up the potential to explore new dimensions of VBDs.

**Application of Machine Learning and Data Integration:** Incorporating machine learning and data integration methodologies provides a promising avenue to harness the vast potential of artificial feeding data in VBD research. Given the complexity of vector–pathogen interactions and the multifaceted nature of the data generated by artificial feeding systems, innovative approaches are crucial for extracting meaningful insights. Machine learning algorithms excel at identifying patterns and correlations within intricate datasets, revealing hidden relationships that conventional analyses of host–vector feeding might overlook [189,190]. Integrating artificial feeding data with genomics, host immunity profiles, and environmental variables allows for the deciphering of the intricate networks governing vector behaviors and pathogen dynamics, enhancing both understanding and predictive capabilities. The synergy between artificial intelligence and artificial feeding systems establishes a positive feedback loop: feeding data refines machine learning models, deepening insights into vector behaviors and pathogen dynamics [190,191]. Data-driven methodologies streamline experimental setups, bolstering research efficiency and reproducibility. As the fields of machine learning and data integration continue to evolve, they present an opportunity to maximize the utility of artificial feeding systems across diverse research domains. These interdisciplinary approaches will propel artificial feeding experiments into a trajectory of data-driven discovery, expediting the development of novel interventions and strategies to mitigate the burden of VBDs.

## 6. Conclusions

In the realm of vector-borne disease studies, artificial feeding systems have evolved into indispensable tools for examining the feeding behaviors, host preferences, and transmission dynamics of pathogen-carrying vectors. Reducing the dependence on live hosts not only minimizes ethical concerns but also mitigates potential risks associated with working with infected animals. The continuous improvement and refinement of these systems will enhance studies aimed at combating VBDs, contributing to the safeguarding of public health on a global scale.

## Data Availability

No new data were created or analyzed in this study. Data sharing is not applicable to this article.
